# Supracolloidal Atomium

**DOI:** 10.1021/acsnano.0c06764

**Published:** 2020-11-11

**Authors:** Jacopo Cautela, Björn Stenqvist, Karin Schillén, Domagoj Belić, Linda K. Månsson, Fabian Hagemans, Maximilian Seuss, Andreas Fery, Jérôme J. Crassous, Luciano Galantini

**Affiliations:** † Department of Chemistry, Sapienza University of Rome, I-00185 Rome, Italy; ‡ Division of Physical Chemistry, Department of Chemistry, Lund University, P.O. Box 124, SE-221 00 Lund, Sweden; § Institute of Physical Chemistry, RWTH Aachen University, DE-52056 Aachen, Germany; ∥ Leibniz-Institut für Polymerforschung e.V. Institut für Physikalische Chemie und Physik der Polymere, DE-01069 Dresden, Germany; ⊥ JARA-SOFT, 52056 Aachen, Germany

**Keywords:** supracolloidal, supramolecular, bile salts, PNIPAM microgel particles, hierarchical self-assembly

## Abstract

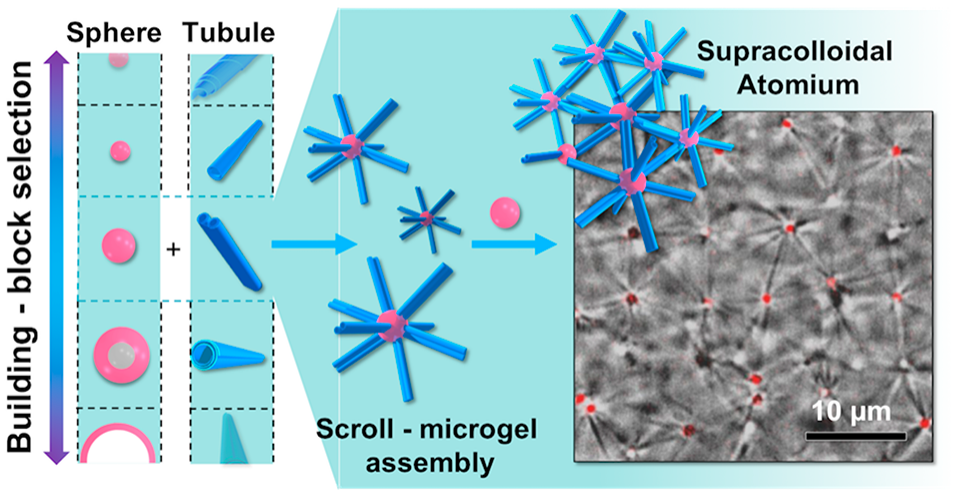

Nature suggests that complex materials result from a hierarchical organization of matter at different length scales. At the nano- and micrometer scale, macromolecules and supramolecular aggregates spontaneously assemble into supracolloidal structures whose complexity is given by the coexistence of various colloidal entities and the specific interactions between them. Here, we demonstrate how such control can be implemented by engineering specially customized bile salt derivative-based supramolecular tubules that exhibit a highly specific interaction with polymeric microgel spheres at their extremities thanks to their scroll-like structure. This design allows for hierarchical supracolloidal self-assembly of microgels and supramolecular scrolls into a regular framework of “nodes” and “linkers”. The supramolecular assembly into scrolls can be triggered by pH and temperature, thereby providing the whole supracolloidal system with interesting stimuli-responsive properties. A colloidal smart assembly is embodied with features of center-linker frameworks as those found in molecular metal–organic frameworks and in structures engineered at human scale, masterfully represented by the Atomium in Bruxelles.

Modern technology strives for control over the assembly in a wide range of length scales, from the molecular to human level, in order to tailor the intimate nano- and microstructure of materials and to optimize their performances in the application. The self-assembly of molecules can be directed in a large variety of geometries due to their complex structure and ability to exploit different intermolecular forces.^1^ Fewer geometries, which are mostly related to compact packing, are obtained in the case of colloidal particles, as a consequence of the limited number of regular shapes available at this scale and the difficulty in directing the interaction to specific binding sites.^2^ The formation of ordered superstructures becomes even more difficult when mixtures of particles of different shapes and length scales are considered.^3,4^ Despite this limitation, anisotropic particles could be cleverly employed as linkers among spherical nodes in bottom-up nanotechnology. Frameworks formed by nodes and linkers can be created at molecular scale by exploiting the directional interactions between atoms and molecules. As examples, metal−organic frameworks,^5,6^ where metal ions or clusters are joined by organic linkers *via* coordination bonds, are currently developed with respect to their great potential in various applications such as gas storage,^7^ separation,^8^ and catalysis.^9^ Similar organizations are very common in structural engineering at human scale, where frameworks in constructions often consist of nodes and linkers designed to withstand high stresses and large pressures. The appealing symmetry of these architectures is well shown in the Atomium monument in Bruxelles, where a framework of spherical nodes and linkers inspired by the atomic ordering has become a central masterpiece. At the colloidal level, the spontaneous formation of such structures is a great challenge as it involves colloids presenting radically different sizes and shapes, interacting *via* highly specific and ideally tunable directional interactions. The most prominent strategy involves complementary DNA bricks^10,11^ or DNA functionalized colloidal building blocks.^12^ Such an approach was proven to be extremely powerful and can be seen as a puzzle where building blocks are able to find each other. Lock and key principles can be also involved, when particle shape complementarity provides reciprocal recognition between the puzzle pieces. However, these methods imply a great synthetic effort to design each piece and lacks flexibility as the specificity is increased, considering that each building block is created for a specific type of assembly. Another concept, that we are following in this study, is the building kit where each building block can be used to create diverse structures. By varying the size/shape and interactions between the different colloidal building blocks, structures can be created including (adaptive) colloidal molecules^13,14^ and crystalline organizations.^15,16^

By taking advantage of the specific amphiphilic structure of natural steroidal surfactants like the bile salts (BSs)^17^ it is possible to rationally design BS derivatives (BSDs) in order to direct their supramolecular self-assembly into tubular structures.^18−22^ These structures provide anisotropic building blocks with selective sites for the binding of polymeric microgels, which can be used to fabricate hierarchical frameworks.

## Results and Discussion

### Supramolecular Linker Design

**Figure 1:**
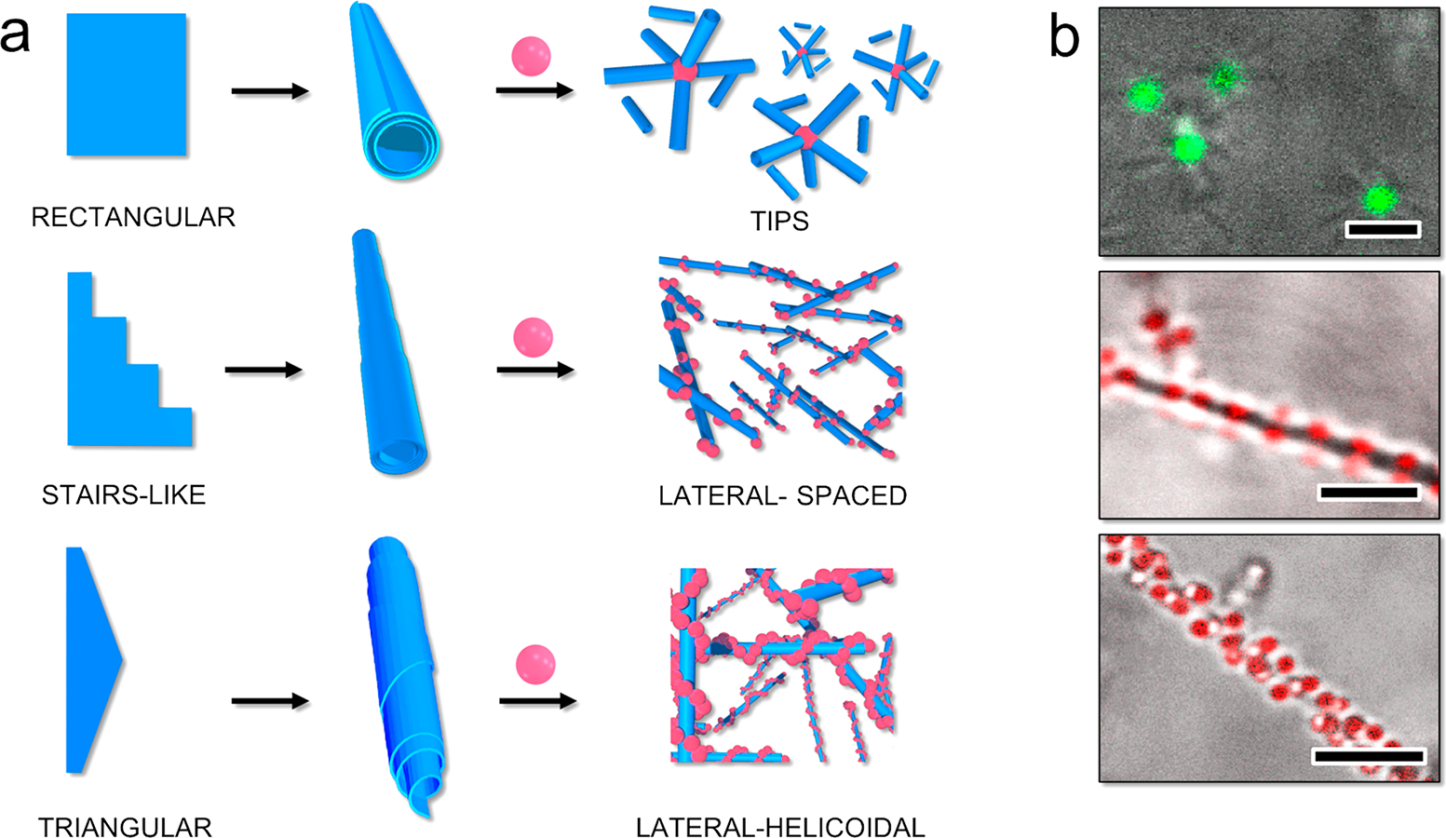
(a) Sketches reporting different hierarchical supracolloidal frameworks achievable between BSD rolls and polymeric microgels. Catanionic mixtures of BSDs at different molar ratios are able to self-assemble into rolled, differently shaped, supramolecular sheets with linear, telescopic, and helicoidal profiles of the external edges. The accessible profiles of the edges direct the interaction with microgels leading to the formation of different superstructures like a microgel decorated with rolls (top panel) and spaced or helically microgel patterned rolls (middle and bottom panels, respectively). (b) CSLM micrographs corresponding to the superstructures schematized on the left given by supramolecular rolls of an anionic BSD in 30 mM Na_2_CO_3_/NaHCO_3_ buffer forming microgel-roll assemblies with positively charged microgels at 2.6 × 10^–2^ wt % (top); supramolecular rolls of a catanionic mixture of BSDs forming rim spaced (middle panel) or helical (bottom) patterns on the roll surface with oppositely charged microgels at 1 × 10^–1^ wt %. Scale bar 2 μm. (b) Bottom panel adapted with permission from ref (23). Copyright 2018 Wiley.

Tubular structures are formed by chemical modifications of the BS amphiphilic distribution that drives the self-assembly into rolled supramolecular sheets. These rolls show specific patterns at the sheet edges on the outer surface depending on the folded sheet shape (Figure 1a).

**Figure 2:**
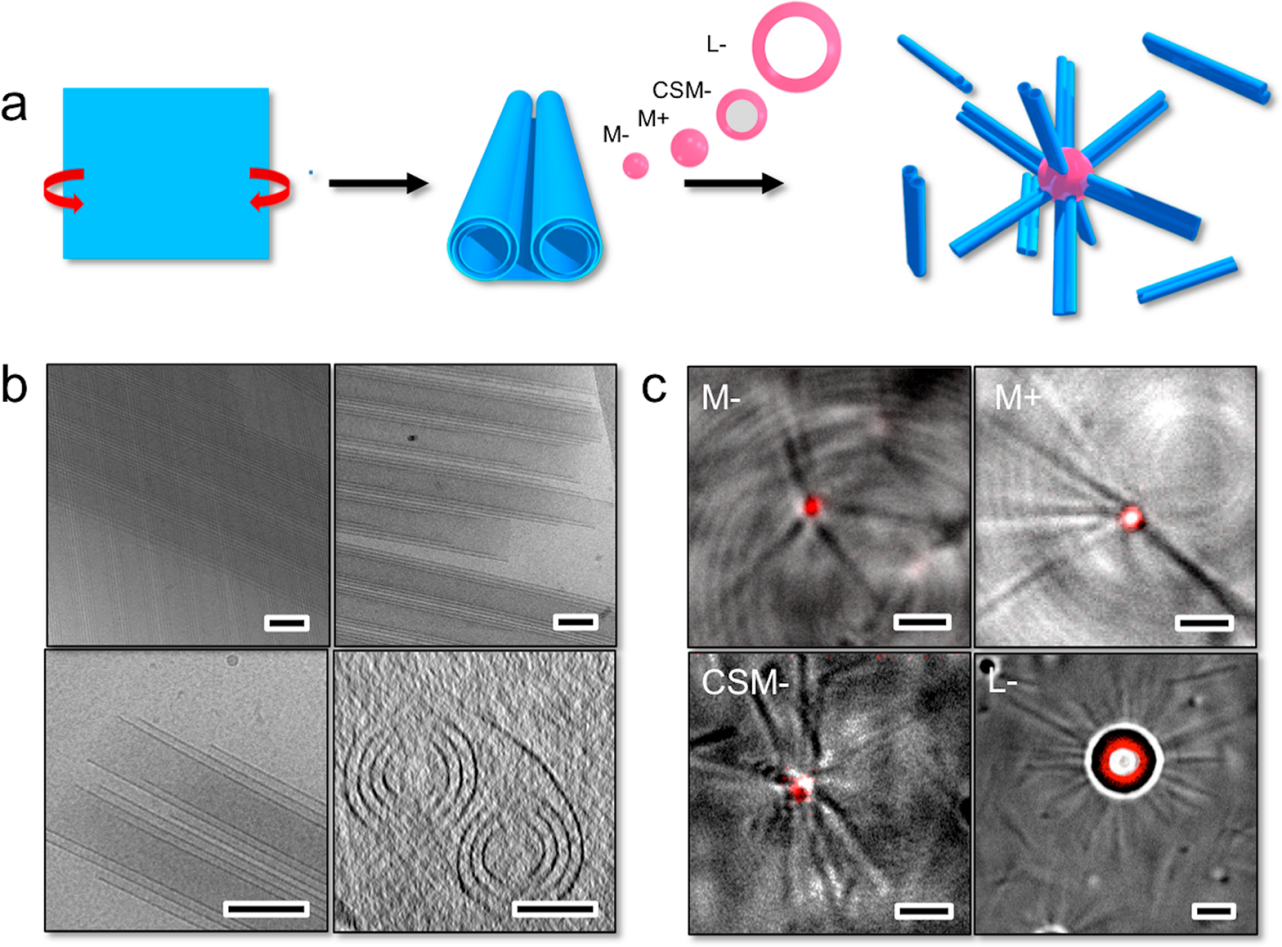
(a) Scheme illustrating the use of NaNAMC supramolecular scrolls to direct the interaction of fluorescently labeled spherical particles of different size and chemical composition toward the extremities and to build a colloidal complex with a central spherical particle and a corona of coordinated scrolls. (b) Cryo-TEM micrographs of NaNAMC 2.0 mM at pH 11.5 highlighting the scroll-like structures and cryo-tomogram (bottom right) showing the cross-section of the scroll (scale bar 100 nm). (c) CLSM micrographs of the scroll-decorated spherical particles (scale bar 2 μm).

In a previous study,^23^ it was demonstrated that a selective association (or binding) of microgels can occur at the bilayer edges located at the extremities and along the lateral surface of the supramolecular rolls. A preferential association takes place at the extremities, where the geometric surface of the spherical microgels can access a longer segment of the edges, resulting in microgels decorated by a corona of rolls (Figure 1b, top). However, since the less preferential binding sites on the lateral surface compete with the extremities, an exclusive association at the ends of the rolls can be accomplished only in the case of weak interaction conditions (*i.e.*, low electrostatic contribution) or at very low fractions of microgels and large excess of tubules. Conversely, when microgels are in excess with respect to the tubules, fully decorated rolls are observed where microgels distribute according to regular patterns following the profiles of the rolled sheet edges (Figure 1b middle, bottom). Interactions at the rims could have multiple origins including both electrostatic and hydrophobic contributions. Hydrophobic interactions are complex in nature and involve many factors such as the hydration energy, surface tension, and elastic modulus.^33^ Many parameters are unknown in our system that is also particularly complex because it involves two different components, the microgels and the scrolls, and it has to account for the topology of the tubule. A simple model based on attractive electrostatic interactions could be used to compare the binding of spheres to oppositely charged tubules having a uniform distribution of the charge or having charges concentrated on a line (see Supporting Information). The comparison allowed us to stress the importance of the rim topology on the specificity of the interactions and to demonstrate a specific binding at the tip of the tubular structures (Figure S1). Selective association at the extremities of the tubular structure is achieved by taking advantage of the ability of a naphthoylamine substituted sodium cholate NaNAMC (Figure S2) to self-assemble into supramolecular scrolls at pH values between 10 and 12. The latter are formed by supramolecular sheets rolled by two sides resulting in paired oppositely wrapped rolls (Figure 2a). They are negatively charged as shown by the electrophoretic mobility value of −4.8 ± 0.7 × 10^–8^ m^2^/(V s), measured on a 2.0 mM NaNAMC scroll dispersion, which allows them to remain well separated in water. Cryogenic transmission electron microscopy (cryo-TEM) and electron tomography (cryo-ET) analyses (see the Experimental Section in Supporting Information) showed that each of the coupled rolls is formed by three to four windings with a spacing between overlapped layers of 11 ± 1 nm and internal and external diameters of 53 ± 3 and 100 ± 4 nm, respectively (Figures 2b, S3, and S4 and video V1). This structure results in a monodisperse maximum transversal distance of the scroll of 200 nm. Furthermore, cryo-TEM and optical microscopy showed that the scrolls exhibit a discrete length with a narrow distribution. An average value of 7.0 ± 1.5 μm was determined by cryo-TEM (Figure S5). Due to their architecture, the scrolls have accessible segments of the supramolecular sheet edges only at the tips as the lateral rims located in the interior of the tubules become inaccessible to large particles. Thus, microgels are expected to bind exclusively at the scroll extremities (Figure 2a). Considering their topology and low polydispersity, these scrolls are expected to present more specific interactions with microgels as compared, for instance, to catanionic tubules and to meet the requirements to build up much more defined assemblies. In the following, these scrolls were therefore tested for the formation of complexes with different colloidal microspheres to elucidate the nature of their interactions and further used as linkers for the achievement of supramolecular networks.

### Microgel–Scroll Complexes

We investigated the association of the tailored BSDs scrolls with various spherical particles comprising homogeneous poly(*N*-isopropylacrylamide) positive (M+) and negative (M−) microgels, core–shell based negative microgels (CSM−) consisting of a polystyrene (PS) core and a shell of poly(*N*-isopropylmetacrylamide) (PNIPMAM) and negative fluorescent hollow PS latex microparticles (L*−*) functionalized with polyvinylpyrrolidone (PVP). The main features of the spherical particles used are summarized in the Table S1. According to previous dynamic light scattering (DLS) and transmission electron microscopy measurements, the different microgels are monodisperse and with hydrodynamic radii (*R*_H_) ranging from 244 to 528 nm at 20 °C. The L− particles are monodisperse as well, with an average overall radius of 1.53 ± 0.05 μm and cavity radius of 0.91 ± 0.05 μm, inferred from confocal laser scanning microscopy (CLSM) statistical analysis (Figure S6). Fluorescent, carboxylated PS microspheres and silica beads with a smooth surface were also used to investigate the contribution of the surface architecture to the sphere–scroll interaction. Details on the synthesis and characterization of L– and silica particles are provided in the Experimental Section of the Supporting Information.

The association between the microgels and latex particles was investigated with an excess of scrolls, which was accomplished in samples at a particle wt % of 8.0 × 10^–4^ (M–, M+), 4.0 × 10^–3^ (CSM−), and 5.0 × 10^–2^ (L−) and a NaNAMC concentration of 2.0 mM (see the Experimental Section of the Supporting Information). Under these conditions, the interaction with the spherical particles, which was strictly directed at the scroll extremities, led to the orthogonal physisorption of the scrolls on the spherical particle surface to form supracolloidal complexes comprising a central microgel that coordinates several scrolls. An overview of the CLSM images of the complexes is reported in Figure 2c. The central particles were evidenced through their fluorescent emission stemming from their covalent labeling with rhodamine B, whereas the scrolls were visible from the transmission measurements. The results demonstrated the specific adsorption of the scrolls with no regard for the chemical polymeric composition and charge of the microgels or whether hollow PS–PVP coated latex were used. A closer inspection of the micrographs showed that the number of adsorbed scrolls increased with the size of the particles (Figure 2c). The exact number could not be determined experimentally; however, we could observe that four or a few more scrolls adsorbed on the smallest microgels (M−, *R*_H_ = 244 nm) with a regular angular spacing. At intermediate microgel size (M+, *R*_H_ = 299 nm), about 6–12 scrolls were found to adsorb per particle. This number of adsorbed scrolls increased further in the case of the core–shell microgels (CSM–, *R*_H_ = 528 nm), and ultimately, a crowded corona of scrolls could be observed to cover the large PS–PVP microparticles (video V2). In all of the samples, the microgel–scroll complexes were homogeneously distributed through the medium and showed similar coordination number and overall size. It is worth noting that the scrolls seemed to associate irreversibly to the microgels. Yet they were found to diffuse on the surface of the particles as observed from their uncorrelated motion, excluding the fact that the apparent scroll-displacement was only the consequence of the rotational diffusive motion of the whole assembly (video V3). Based on geometrical considerations, the theoretical maximum number of packable scrolls attached onto the microgel surface can be estimated as , where *D*_T_ is the diameter of one of the two coupled rolls forming the scroll. The equation is valid assuming thin rolls, for which the microgel surface can locally be approximated as flat, and a prefactor  corresponding to the maximum 2D disk (roll cross section) close packing ratio. By assuming free rotation along the long axis of the scroll this number can be reduced to  , *i.e.*, by a factor of 0.5. This implies that the average number of tubules would be reasonably between 7.5 and 15.0 for M– and between 32.5 and 65.0 for M+. Still, such estimation does not account for curvature effect, microgel softness, and the specific interactions of the scrolls and thus only provides a rough estimate of the maximum number of adsorbed tubules. These numbers are consistent with the observations, considering that close packing is hindered by electrostatic repulsion between the scrolls that are negatively charged and that the additional lateral mobility of the scrolls entails a looser packing at the microgel surface.

As both positive and negative microgels were able to form the complexes, we can conclude that the electrostatic interactions were not relevant to the microgel–scroll binding. Electrostatics may have played an important role in our former study,^23^ considering that in most of the cases the low ionic strength of the dispersions was investigated. However, for the NaNAMC scrolls we could observe some association independent of the nature of the charge (positive or negative) of the linkers. This is certainly related to the relative high salinity of the buffer solutions and the related screening of the electrostatic interactions. As such we expect hydrophobic interactions to prevail. The latter are related to the increased exposure of the molecules at the tubule rims.

Further experiments showed in addition that no interaction occurred between scrolls and smooth PS spheres or silica beads, as presented in video V4. These results suggests that the loose polymeric texture of the microgels crucially contributes to the sphere−scroll binding, which plausibly stems from hydrophobic interactions involving the rims of the scroll forming bilayer of BS molecules, accessible at the scroll extremities, and the dangling chains belonging to the peripheric soft deformable network of the microgels. The protrusion of these chains into the fluid lipid bilayer was recently used to justify the association of microgels with giant unilamellar vesicles consisting of a fluid lipid bilayer.^24^ In addition, the loose external network of the microgel allows the outer surface of the particles to adapt to the irregular extremities of the scrolls (Figure 2b), thereby optimizing the interaction with the rims.

### Three-Dimensional Framework

**Figure 3:**
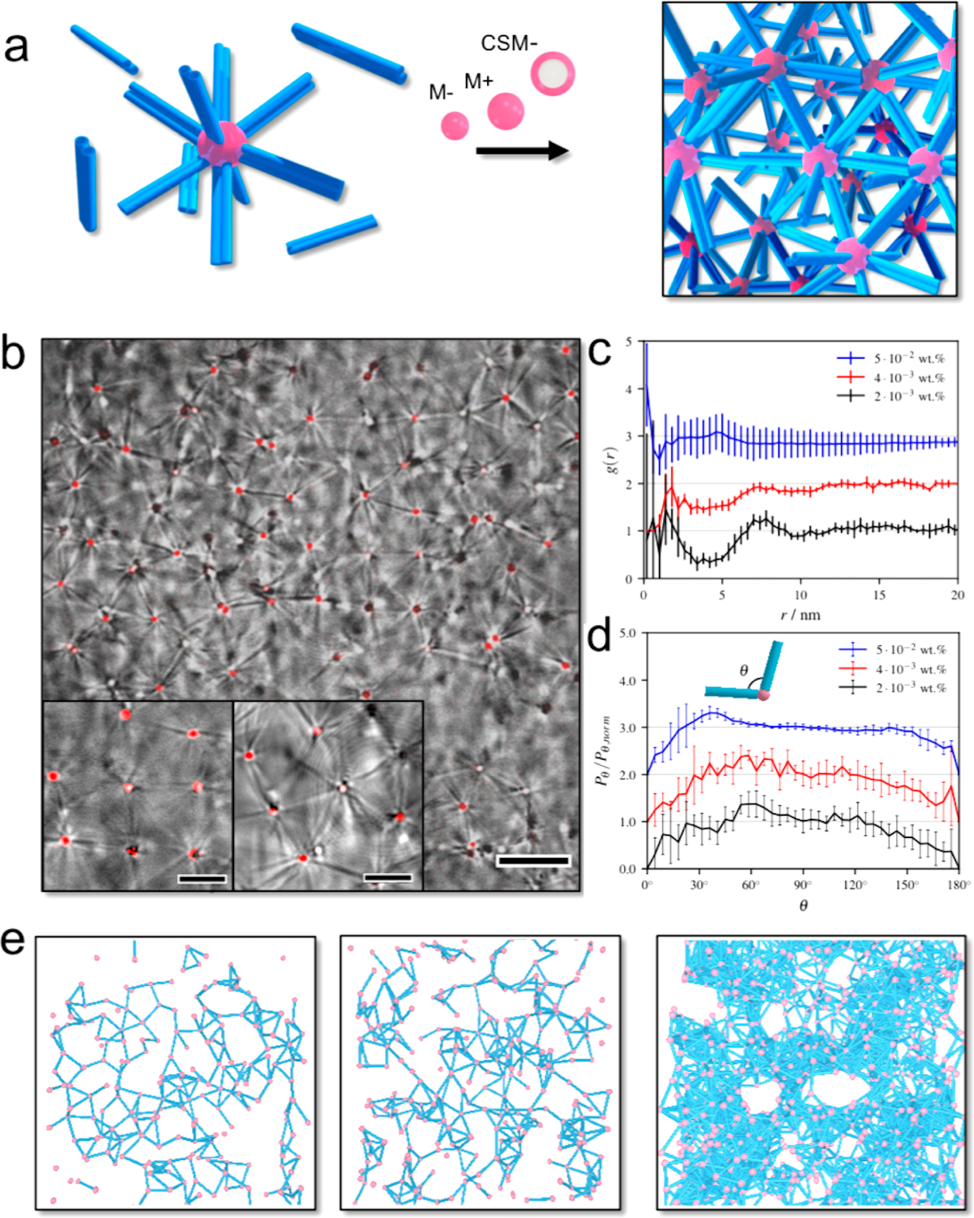
(a) Sketch representing the supracolloidal framework formation by mixing preformed NaNAMC scrolls with fluorescently labeled spherical particles. (b) CLSM micrographs of a mixture of *c*_μgel_ = 2.0 × 10^–3^ wt % M+ microgel suspension with supramolecular NaNAMC scrolls at 2.0 mM NaNAMC concentration and pH 11.5. Scale bar 10 μm, inset 5 μm. (c) Corresponding 3D *g*(r) of the framework extracted from acquisition of *z*-stack images obtained through CSLM. (d) Angular distribution between connecting tubules as a function of the microgel concentration. (e) 3D reconstruction of the supracolloidal framework at microgel concentration of *c*_μgel_ = 2.0 × 10^–3^, 4.0 × 10^–3^, and 5.0 × 10^–2^ wt % (from left to right).

The complexes formed by coassembly of scrolls and microgels were stable and well separated in samples at low microgel/scroll number ratios. From these conditions, we observed that the increase of the microgel fraction might reduce the average number of tubules associated with each microgel and lead to a spontaneous hierarchical coassembly into interconnected frameworks (Figure 3a).

The frameworks reproduce at the colloidal level the architecture of spheres connected by poles of the Atomium or the “stick-and-ball” models of molecules, where microgels play the role of atoms and the scrolls that of bonds (video V5). The ability of these complexes to interconnect into a framework was related to the highly specific interactions of the microgel at the extremities of the scrolls.

With this knowledge, the microgel concentration (*c*_μgel_) was systematically varied for M+ to determine the optimal conditions for an extended framework formation, keeping the NaNAMC concentration constant at 2.0 mM and the pH of the suspension at 11.5. Starting from the dispersions of well-separated microgel–scroll complexes at *c*_μgel_ = 8.0 × 10^–4^ wt %, we observed that frameworks started to arrange in clusters with the progressive increase of microgel fraction until a full spanning three-dimensional framework formed at *c*_μgel_ = 2.0 × 10^–3^ wt % (Figures 3b and S7 and videos V6 and V7). Details on the supracolloidal structures provided by the CLSM micrographs show that several scrolls belonging to a central microgel were involved in the coordination of the nearby microgels, and coordination numbers of 4–6 on the focal plane were identified. No free scrolls were found throughout the whole volume investigated; consequently, they were all engaged in the interaction with microgels. Although the large majority of scrolls were stuck in the interconnections of different microgels, some of them were sometimes found to be adsorbed to the microgels by only one extremity, preserving their diffusional motion at the microgel surface. This supports the statement that the scrolls were not fixed on the microgel surface but could move over it (video V8). The framework formation was associated with a long-range spatial correlation between the interconnected microgels (Figure 3b), which could be analyzed by estimating the 3D radial distribution function *g*(r) of the microgels. The analysis performed at different *c*_μgel_ (Figure 3c) allowed us to qualitatively infer the effect of microgel/scroll stoichiometry on the framework order. At *c*_μgel_ = 2.0 × 10^–3^ wt %, the main correlation peak was found around 7.5 μm, which is consistent with interparticle distances comparable to the scroll length. Interestingly, the formation of regular geometries such as squares or triangles was sometimes locally observed (Figure 3b insets). By increasing *c*_μgel_ to 4.0 × 10^–3^ wt %, the selective binding at the tubular tips was preserved, but the structural order was lowered, as reflected by the decrease of the *g*(r) correlation peak. At *c*_μgel_ = 5.0 × 10^–2^ wt %, a further lowering of the order was detected and a detrimental effect on the framework connectivity was visually appreciable (video V9). Under these conditions, complexes started to build up in which scrolls were observed to bind two or more microgels on each tip.

The angular distribution between connecting scrolls was analyzed as well (Figure 3d). For this purpose, we relied on the spatial distribution of the imaged microgels and reconstructed the 3D framework assuming the presence of bonding scrolls whenever two microgels were positioned at a cutoff distance between 6 and 8 μm (Figure 3e and video V10). Considering the angle θ formed by two connecting scrolls adsorbed to the same microgel, the angular distribution was sampled for different *c*_μgel_ and reported as *P*_*θ*_/*P*_θ,norm_, *i.e.*, the probability of an angle θ (*P*_*θ*_) normalized to a uniform probability distribution (*P*_θ,norm_). Under optimal conditions at *c*_μgel_ = 2.0 × 10^–3^ wt %, *P*_*θ*_/*P*_θ,norm_ showed a maximum at θ around 60° indicating the formation of local triangular lattices. Yet no angular wide-range ordering was identified considering the absence of peaks at θ = 120° and 180°. Similar results were found for *c*_μgel_ = 4.0 × 10^–3^ wt %, at which the *g*(r) analysis showed that the framework was preserved to a large extent. However, for *c*_μgel_ = 5.0 × 10^–2^ wt %, significant changes were visible with a shift of the peak to 36°. Under these conditions, the framework was much less interconnected and more heterogeneous with dense local microgel/tubule aggregates, with more than one microgel located at scroll extremities. We anticipate that a more compact structure of these aggregates, compared to the framework, dictated the peak shift.

Negatively charged microgels CSM– and M– were also used to obtain supracolloidal frameworks, whose order was observed to be dependent on the microgel sizes. As shown by the corresponding *g*(r) function, the microgel–microgel correlation was significantly more promoted in the case of the microgel with the smaller size M– (*R*_H_ = 244 nm) than for the larger CSM– (*R*_H_ = 528 nm) (Figure S8). These experiments indicated that the relative size of scrolls and microgels, along with their number ratio, affected the framework structure by setting the valence of the microgel nodes. Assuming that the charge of the microgel was not relevant for the scroll binding, we can conclude that an optimal balance of these parameters occurred for M+ in the sample at *c*_μgel_ = 2.0 × 10^–3^ wt % and 2.0 mM NaNAMC, for which the supracolloidal framework was the most defined (Figure S8).

The framework formation after mixing involved a progressive slowing down of its dynamics with time. This was reflected in a gradual decrease in the relaxation time of the scattered intensity autocorrelation functions inferred from DLS measurements (Figure S9a).^29^ After about 2 h 50 min, an equilibrium value in relaxation time was reached, which was longer than those of the microgels or the tubules separately (Figure S9b). The progressive slowing down of the diffusion process could be observed also through CLSM, showing that as the supracolloidal framework gradually formed microgels reduced their motion around their average position (video V11).

The scrolls are sensitive to both temperature and pH. By increasing the temperature, they break at a biologically relevant critical value around 30–35 °C and reversibly reform at the same temperature by cooling. This behavior could be monitored by light scattering (Figure S10) and circular dichroism (CD) measurements, as the scroll formation leads to an increase of the scattered light intensity and at the same time the appearance of a typical CD signal, ascribed to the helical supramolecular arrangement of the scroll forming NaNAMC molecules.^19^ Likewise, CD and light scattering measurements demonstrate that, starting from the working pH 11.5, scrolls disappear by lowering the pH below a critical value of about 9.5 and reversibly reform by reproducing the original more alkaline conditions. The pH- and thermo-responsiveness of the scrolls impart a combined responsiveness to the whole framework. To verify these properties, a sample of well-formed framework at 20 °C and pH 11.5 was heated to 45 °C and then cooled to the original temperature. DLS, CLSM, and CD measurements revealed that the framework was destroyed by heating, due to the scroll disassembly, and reformed by cooling the sample, as a result of the scroll reassembly (Figures S11 and S12 and video V12). The temperature scan performed by DLS, in particular, allowed us to appreciate an abrupt change of the relaxation time of the autocorrelation function around 30 °C, where the destruction and reformation of the framework occurred (Figure S11a). We observed by CLSM that the reformed framework after a scan up and down in temperature was slightly less ordered compared to the original framework, as demonstrated also by the estimated *g*(r) (Figure S13). The lower framework order was due to a larger polydispersity of the lengths of the supramolecular scrolls, when reformed after the temperature change. Likewise, as shown by DLS (Figure S14) and CLSM (video V13) the framework broke and reversibly reformed by lowering the pH from 11.5 to 7.5 and then increasing it back to the original more alkaline value. The order was more preserved when the framework reformed after the pH change (Figure S15). In any case, both after the pH and temperature scans, the connectivity of the framework was fully reformed and no free tubules were observed within the investigate volume (videos V12 and V13).

## Conclusion

Spontaneous formation of lattices consisting of nodes interconnected by linkers occurs at the molecular level by exploiting the ability of central atoms to form complex with multifunctional ligands to provide frameworks with large application potential. The formation of similar complexes and frameworks at supracolloidal level requires building blocks with different shapes, like spheres and rodlike linkers, and a carefully directed binding of the spherical nodes to the tips of the rods. In this work, these conditions have been implemented by using polymeric spherical particles and supramolecular scrolls built up of a bile salt derivative. The results demonstrate that a high scroll–microgel interaction specificity allows for the spontaneous formation of well-defined scroll–microgel complexes and ultimately, under suitable conditions, an extended regular framework. These frameworks offer colloidal scale analogues of metal–organic frameworks at a molecular scale, where a fine-tuning of the interactions is implemented by the topology of the linker, by reproducing Atomium-like macroscopic architectures. Colloidal networks of supramolecular microtubules and fibrils, like actin, play fundamental roles in biology, *e.g.*, in regulating the mechanical features of cells and in driving intracellular signaling. Inspired by these concepts the reported Atomium-like colloidal frameworks provide bases for rational fabrication of functional networks with specific mechanical response, channel protected internode exchange of signaling materials and easy tunability *via* external stimuli. Future functional frameworks can be further envisioned whose features can be modulated by changing the dimension and physical nature of colloidal nodes and linkers.

## Materials and Methods

### Synthesis

#### Bile Salt Derivative Naphthoylamine Substituted Sodium Cholate (NaNAMC)

The synthesis started from cholic acid that was first converted into a cholic acid ester and then transformed into the corresponding 3β-amino substituted derivative *via* Mitsunobu reaction with diphenylphosphoril azide and Staudinger reduction with PPh_3_/H_2_O of the intermediate azido compound in THF.^25^ The BSD NaNAMC was obtained by reacting naphthoyl chloride with the 3β-amino-substituted cholic ester as described in former studies.^19,20^

#### Microgel Synthesis

Poly(*N*-isopropylacrylamide) M– and M+ microgels were synthesized by precipitation polymerization as described in our previous works.^23,26,27^ The microgel charge stems from the initiator employed during the synthesis; *i.e.*, anionic microgels were synthesized using potassium persulfate as initiator and cationic microgels using 2,2′-azobis(2-methylpropionamidine) dihydrochloride as initiator. The poly(styrene)/poly(*N*-isopropylmethacrylamide) core–shell particles CSM– were synthesized in a two-step reaction as described in a previous study.^27^ The cross-linking degree of all microgels was ensured by copolymerization with *N*,*N′*-methylenebis(acrylamide) cross-linker (5 mol % in respect to *N*-isopropylacrylamide or *N*-isopropylmethacrylamide monomers). The microgels were covalently labeled using methacryloxyethylthiocarbomoyl rhodamine B dye during their synthesis. We refer to our former studies for more details on the microgel synthesis and characterization. The hollow particles were synthesized *via* dispersion polymerization following a procedure reported in the literature.^28^

#### Latex Particles

A full description of the PS–PVP-coated hollow particles (L−) and silica beads synthesis is reported in Supporting Information.

### Sample Preparation

For the scroll preparation, the pH of a water solution containing the BSD NaNAMC (concentration 2 mM) was raised dropwise to pH 11.5 through addition of NaOH 0.2 M. The final solution was characterized by visual turbidity effect and birefringence due to the presence of the tubular nanostructures. Sample was then sealed and stored at 20 °C for 3–4 h. Scroll–microgel complexes were then prepared by mixing dropwise a suspension of particles in a 30 mM HCO_3_^–^/CO_3_^2–^ buffer solution to the previous tubule dispersion at pH 11.5. The mixing was accomplished by injecting the microgel directly into the tubule dispersion through a syringe connected to the sample vial through tubing, while the sample vial was maintained under gentle stirring with a magnetic stirrer.

### Confocal Laser Scanning Microscopy

A Leica SP5 confocal laser scanning microscope (D6000I) using a 100 × /1.4 NA oil immersion objective in the inverted mode was used for image acquisition. As light sources, two emission lines at 488 and 543 nm from an argon and a He–Ne laser respectively, were used. The intensity of the lasers was kept always around 30–35% while the detection bandwidths were set between 500 and 574 nm for fluorescein dye emission detection and between 555 and 650 nm for the rhodamine B dye emission detection. Pinhole size was set at 1 AU (151.5 μm). The microscope was mounted in a temperature controlled enclosure providing a temperature stability of ±0.2 °C. The samples were kept between two cover glasses separated by a 0.12 mm spacer (Invitrogen Secure-Seal imaging spacer). Temperature controlled measurements of cooling and heating were performed waiting an equilibration time of 10 min in order to ensure the equilibration of the specimen with the enclosure temperature. The acquisition software used was Leica Application Suite Advanced Fluorescence (LAS AF) supplied from Leica. All the micrographs reported are displayed as the superimposition of the fluorescence emission (red or green) and the transmission (gray scale background) channels of microscope laser. In several cases, channels were slightly enhanced in contrast or/and luminosity for the sake of clarity. Fluorescence emission data were not used for quantitative analysis purposes. Detailed acquisition settings for each image are reported in the Supporting Information.

### Radial Distribution Function Calculations

The 3D particle tracking from the ImageJ software (https://imagej.net/Fiji) was used to extract the microgel positions. The radial distribution functions were retrieved by using a self-written code by sampling a histogram of all microgel pair distances and thereafter normalizing each histogram element with the corresponding volume element at that distance and scaling with the number density of microgels. In order to retrieve the volume elements, we randomly positioned a large number of points in an equally sized unit-cell and from there sampled a histogram given these positions. Since the points are uncorrelated, their radial distribution equal unity at every distance, and thus we reversely retrieve the corresponding volume elements. The used data came from {8,5,6} configurations retrieved from experiments with *c*_μgel_ of 2 × 10^–3^, 4 × 10^–3^, and 5 × 10^–2^ wt %, which is the same configuration used when retrieving the angular correlations. The standard deviations were calculated using {8,5,6} configurations for each concentration, respectively.

### Cryo-electron Tomography

For cryo-ET, the samples were prepared by mixing the original NaNAMC suspension with a suspension of gold nanoparticles (5 or 10 nm protein-A gold fiducials, UMC Utrecht, The Netherlands) in a volume ratio of 10:1 just seconds before plunge freezing. This procedure was applied to minimize any effect of the addition of gold nanoparticles on the tubular nanostructure integrity. As verified by cryo-TEM imaging, apart from the presence of gold fiducials, the tubules observed in the cryo-ET samples otherwise appeared indistinguishable from the tubules seen in the control samples. The samples for cryo-ET were prepared using Quantifoil R3.5/1 200 mesh copper TEM grids (Ted Pella, Redding, USA) following the same plunging protocol as in the case of control samples. Tilt series acquisition of zero-loss images was performed on the same JEOL microscope using Serial EM software.^30^ Tilt series were acquired in bidirectional fashion, in steps of 2°, covering the views from −60° to +60°. The electron dose per tilt view was kept below 2.5 e^–^/Å^2^. Tilt series alignments and tomographic reconstructions were done using IMOD.^32^ The tomographic reconstructions (2048 × 2048 × 350 voxels) were obtained using a simultaneous iterative reconstruction technique (SIRT) algorithm after 15 iterations. Final visualization of data was performed in ImageJ software;^31^ to improve the image clarity and reduce the file size, each slice in the final tomogram was created as an average of 10 consecutive slices from the original tomogram.

## Supporting Information Available

Figures S1–S15 showing additional experimental details; Table S1 shoing particle characteristics (PDF)
Analysis of a tubular cross section through cryo-ET (AVI)
Supracolloidal assemblies of spheres and scrolls (AVI)
Details of scroll-decorated M+ microgel assemblies (AVI)
Absence of association between the scrolls and “smooth” spherical particles (AVI)
Supracolloidal cluster formation in a dilute dispersion (AVI)
Analysis of the 3D supracolloidal framework through CLSM (AVI)
3D reconstruction of a supracolloidal framework from CLSM data (AVI)
Free diffusion of the scrolls at the microgel surface (AVI)
Effect of microgel concentration on supracolloidal framework order (AVI)
Reconstruction of 3D framework by computer simulation (AVI)
Progressive slowing down of microgel diffusion in supracolloidal framework with time (AVI)
Thermoresponsiveness of the supracolloidal framework (AVI)
pH responsiveness of the supracolloidal framework (AVI)

## Supplementary Material

nn0c06764_si_001.pdf

nn0c06764_si_002.avi

nn0c06764_si_003.avi

nn0c06764_si_004.avi

nn0c06764_si_005.avi

nn0c06764_si_006.avi

nn0c06764_si_007.avi

nn0c06764_si_008.avi

nn0c06764_si_009.avi

nn0c06764_si_010.avi

nn0c06764_si_011.avi

nn0c06764_si_012.avi

nn0c06764_si_013.avi

nn0c06764_si_014.avi
