# Supplementary material for: Supracolloidal Atomium
Source: ACS Nano. 2020 Nov 11;14(11):15748–56. doi: 10.1021/acsnano.0c06764 (PMC8016364; doi:10.1021/acsnano.0c06764)
Supplement: Supplementary file 1 — nn0c06764_si_001.pdf [file nn0c06764_si_001.pdf]

Supporting Information

Supracolloidal Atomium

Jacopo Cautela, Björn Stenqvist, Karin Schillén, Domagoj Belić, Linda K. Månsson, Fabian Hagemans, Maximilian Seuss, Andreas Fery, Jérôme J. Crassous*, Luciano Galantini*

*Correspondence to: luciano.galantini@uniroma1.it, crassous@pc.rwth-aachen.de

Supplementary Materials and Methods

Synthesis

PS-PVP coated hollow particles (L-) synthesis. The hollow particles were synthesized *via* dispersion polymerization following a procedure reported in the literature (see Ref. 30). 53.1 mg polyvinylpyrrolidone (PVP Mw 55 kDa (approx.), Sigma-Aldrich) and 544.3 mg ammonium persulfate (APS, Sigma-Aldrich) were pre-weighted in a 250 mL jacketed two necks round-bottom flask reactor and dispersed in a mixture of 125 mL ethanol and 15 mL MilliQ water containing 2.8 mg of MRB dye. The aqueous dye solution was sonicated beforehand for 10 min to ensure the proper MRB redispersion. Stirring was ensured by a magnetic stirrer set at sufficient speed to create a large vortex. After 10 min redispersion, 11 mL styrene (BASF), destabilized over Al_2O_3 column were added to the reaction mixture and the dispersion was heated up to 70 °C. The reaction was let to proceed for 48 h. During the reaction, 2 mL aliquots were collected after 4, 8, 12, 16, 21, 24, 28 and 48 h, respectively to follow the transformation of the latex particles from plain to hollow (Figure S6). The formation of hollow particles was rationalized by the fact that the reaction took place at the surface of the particles. However, whereas the hollow particle formation could be reproduced, no consecutive particle buckling into bowl-shaped particles was observed as reported by Park *et al.* Each sample was centrifuged 4 times at 10000 rpm for 2 min, redispersed 2 times in ethanol and 2 times in water. Redispersion after each centrifugation cycle was ensured by 18 min sonication. We finally used the 48 h particles for our experiments considering their large size with a diameter of 3.05 ± 0.10 µm determined by CLSM and their reduced buoyancy stemming from their hollow conformation with a cavity diameter of 1.82 ± 0.11 µm. Their stabilization with relatively large molecular weight PVP is considered in this study to investigate the influence of the surface functionalization on their assembly with the NaNAMC scrolls.

Carboxylated polystyrene latex. Fluorescent carboxylated polystyrene particles with a mean diameter of 500 nm with an excitation wavelength λ_{ex}~520 nm and an emission wavelength, λ_{em}~540 nm were purchased from Sigma (reference L5530).

Silica beads synthesis. The particles were synthesized through a two-step synthesis. In the first step, silica seeds are grown in a typical Stöber growth procedure. In the second step, the particles were grown to the desired size by a seeded Stöber growth method.

Step 1. First, fluorescently labeled silica seeds were prepared. To this extent, 49 mg of fluorescein-isothiocyanate (95%, Sigma-Aldrich) were dissolved in 2 mL of absolute ethanol (VWR). Then, 300 µL of 3-aminopropyltriethoxysilane (99%, Sigma-Aldrich) were added and the mixture was stirred in the dark for 3 h. The silica seeds were then prepared as follows. To a mixture of 330 mL absolute ethanol and 34 mL ammonia (26.1% NH_3 basis, Sigma-Aldrich), 14 mL tetraethylorthosilicate (TEOS, 98%, Sigma-Aldrich) were added while stirring vigorously. After the mixture turned turbid, the stirring was reduced to a moderate speed and continued at this speed for 48 h. The particles were collected by centrifugation (340 G, 25 min) and redispersion in absolute ethanol. Residual dye was removed by additional washing steps with absolute ethanol.
Step 2. To 60 mL absolute ethanol, 7.6 mL (65 g/L) of silica seeds were added and sonicated for 30 min. Sonication reduces the number of dumbbells formed in this step. Then 8.9 mL MilliQ grade water and 3.6 mL ammonia (26.1%) were added. While stirring vigorously, the following solutions were added simultaneously at a constant rate of 0.57 mL/h using a syringe pump. The first solution consists of 20 mL TEOS diluted with 40 mL absolute ethanol. The second solution consists of 41.3 mL absolute ethanol, 13.3 mL MilliQ water and 5.4 mL ammonia (26.1%). The second solution is added to compensate for dilution of water and ammonia by the addition of the first solution. After the last addition, the solution was stirred for an additional 24 h. The particles were collected by centrifugation and redispersion in absolute ethanol (340 G, 25 min). The particles were sedimented overnight and the supernatant, containing the secondary nucleation, was replaced by absolute ethanol. The process was speeded up by spreading the particles over several tubes. This procedure was repeated until all secondary nucleation had been removed, confirmed by electron microscopy. The particles were transferred from ethanol into water *via* centrifugation and sonicated for 30 min to ensure their proper redispersion before mixing with NaNAMC scrolls.

A summary of the microgel based spherical particles and hollow PS/PVP latex characterization is provided in Table S1.

Methods

Confocal Laser Scanning Microcopy images acquisition specific settings. Fig. 1c, top. The micrograph was cropped from a 512x512 px, x81 steps, 31x31x20.17 µm (-*x*, -*y*, -*z*), *z*-stack slice acquired at 5x magnification and 8 bit of logical resolution. Frame and line averaging were set at 2 and 4, respectively. Suspension medium was a 30 mM carbonate buffer water solution at 20°C. *Fig. 1c, middle*. The micrograph was extracted from a 512x256 px, x97 steps, 15.50x7.73x12.10 µm (-*x*, -*y*, -*z*), *z*-stack acquired at 10x magnification and 8 bit of logical resolution. Frame and line averaging were set at 1 and 8, respectively. Suspension medium was water and temperature was set at 20°C. *Fig. 1c, bottom*. The micrograph was cropped from a 512x512 px, x120 steps, 31x31x15 µm (-*x*, -*y*, -*z*), *z*-stack slice acquired at 5x magnification and 8 bit of logical resolution. Frame and line averaging were set at 1 and 4 respectively. Suspension medium was water and temperature was set at 20°C.
Fig. 2c, upper left. The micrograph was extracted from a 512x512 px, x112 steps, 15.50x15.50 µm, x25.483 s (-*x*, -*y*, -*t*), time series acquired at 10x magnification and 8 bit of logical resolution. Frame and line averaging were set at 1 and 7, respectively. Suspension medium was water at pH 11.50 and temperature was set at 20°C. *Fig. 2c, upper right*. The micrograph was extracted from

a 128x128 px, x301 steps, 10.33x10.33 µm, x11.483 s (*-x, -y, -t*), time series acquired at 15x magnification and 8 bit of logical resolution. Frame and line averaging were set at 1 and 4 respectively. Suspension medium was water at pH 11.50 and temperature was set at 20°C. *Fig. 2c bottom left.* The micrograph was extracted from a 512x512 px, x38 steps, 15.50x15.50 µm x4.957 s (*-x, -y, -t*), time series acquired at 10x magnification and 8 bit of logical resolution. Frame and line averaging were set at 1 and 4, respectively. Suspension medium was water at pH 11.50 and temperature was set at 20°C. *Fig. 2c, bottom right.* The micrograph is 512x512 px, 15.50x15.50 µm (*-x, -y*), acquired at 10x magnification and 8 bit of logical resolution. Frame and line averaging were set at 1 and 5 respectively. Suspension medium was water at pH 11.50 and temperature was set at 20°C. All the images extracted from time series are the result of the intensity averaging performed on all the acquired frames of the two channels, separately. *Fig. 3b and Fig. S7a.* Micrographs were extracted from a 1024x1024 px, x81 steps, 77.50x77.50x9.98 µm (*-x, -y, -z*), *z*-stack acquired at 2x magnification and 8 bit of logical resolution, with steps of 0.13 µm. Frame and line averaging were set at 20 and 4 respectively. Suspension medium was water at pH 11.50 and temperature was set at 20°C.

Dynamic Light Scattering. Dynamic light scattering measurements of the scroll-microgel mixtures were performed using an ALV/DLS/SLS compact goniometer system from ALV-GmbH, Langen, Germany (see Ref. 26). The light source is a 22 mW He-Ne Laser operating at 632.8 nm, the intensity of which is modulated using an automated attenuator. A Glan laser polarizer prism put in front of the cell housing ensures vertical polarization of the laser beam. The sample cuvettes are immersed in a thermostated vat filled with a refractive index matching liquid (decalin). In this work, 10 mm-borosilicate glass cuvettes were used. The control of temperature is achieved through a F32 Julabo heating unit (± 0.01 °C). The scattered light is collected using a detection unit that includes a near-monomodal optical fiber and two high-quality avalanche photodiodes. The time correlation function of the scattered intensity $G^{(2)}(t)$ is obtained using an ALV-7004 multiple tau digital correlator. In this work, unpolarized autocorrelation measurements were performed and the normalized functions are presented as $g^{(2)}(t)-1$ *versus* lag time t. Measurements of the 2.0 mM NaNAMC scroll dispersion, of the $5.0 \cdot 10^{-4}$ wt% M+ microgel suspension and of the supracolloidal Atomium framework formed at 2.0 mM NaNAMC and $2.0 \cdot 10^{-3}$ wt% M+ after 1 h of equilibration were compared, setting the scattering angle (θ) to 90°, and acquiring the respective autocorrelation functions of a duration of 120 s. For the measurement of the kinetics of the supracolloidal Atomium formation, 1 mL of 2.0 mM NaNAMC scroll dispersion, 4 h after preparation was pipetted in the cylindrical cuvette and placed in the instrument vat at 20 °C for 1 h of temperature equilibration. Then, 100 µL of a microgel suspension was slowly injected, and the cuvette was gently shaken to favor the mixing prior to the measurement. The final microgel concentration was $2.0 \cdot 10^{-3}$ wt% and the pH 11.5. Time resolved DLS were then performed by acquiring an autocorrelation function for 120 s, at θ = 90° with time interval of 10 min for 2 h and 50 min after mixture preparation. For the measurements of the supracolloidal Atomium thermoresponsive behavior, a glass cuvette containing the supracolloidal Atomium formed in a mixed sample of 2.0 mM NaNAMC scrolls and $2.0 \cdot 10^{-3}$ wt% M+ microgels was placed in the instrument vat at 20 °C, left to equilibrate for 1 h and then measured. Thereafter, the temperature was increased at 45 °C and left to equilibrate for 45 min in order to ensure the complete disaggregation of the self-assembled BSD scrolls. Then a temperature scan was performed by

cooling the sample down to 20 °C in increments of *ca.* 1 °C and by acquiring at each step the autocorrelation functions at θ = 90° for 60 s, after a temperature equilibration time of 10 min. For the measurements of the pH responsive behavior, a sample of supracolloidal Atomium, formed in a mixture of 2.0 mM NaNAMC and 2.0 · 10^{-3} wt% microgels, was equilibrated in the instrument vat at 20 °C and pH 11.5, for 1 h, prior to the measurement. Subsequently, the sample was extracted from the cuvette and transferred into a vial. The pH value of the mixture was then brought to pH 7.5 by controlled addition of 0.1 M HCl. The sample once transferred into the cuvette again and placed back in the vat, and its correlation function was measured after temperature equilibration. Finally, in the same manner, the pH of the sample was increased to 11.5 by controlled addition of 0.1 M NaOH and measured. The light scattering intensities of the temperature induced disaggregation and reformation of the scrolls at 2.0 mM NaNAMC and pH 11.5 were extracted from DLS measurements performed in a Malvern Zetasizer Nano ZS equipped with a He-Ne laser, working in backscattering mode (θ = 173°) and with an automated attenuator. Prior to the measurements, the sample was placed in a PMMA cuvette and equilibrated for 20 min at 20 °C in the instrument cell holder, whereafter temperature scans were performed in which the intensity autocorrelation function was acquired with increments of 2 °C, heating the sample from 20 °C to 42 °C and then cooling back to 20 °C, with an equilibration time of 10 min at each step. The reported relaxation time (τ) is the result of the average of three consecutive measurements with the duration time of 60s.

Electrophoretic mobility measurements. A Malvern Zetasizer Nano-ZS Series was used for the electrophoretic mobility measurements. The instrument was equipped with a He-Ne laser and an automated intensity attenuator. The measurements were carried out at 20 °C on diluted microparticle suspensions (<0.1 wt%) using Malvern disposable folded capillary cells DTS1070. The reported zeta potential values reported are stated as the average of 20 consecutive measurements.

Circular Dichroism spectroscopy. A JASCO model 715 spectrophotopolarimeter was used for the circular dichroism (CD) and UV spectra acquisition. Spectra were recorded by using quartz cuvettes with path lengths of 0.1 mm. After subtraction of the cuvette and solvent contribution to the absorption, the CD curves were reported in molar ellipticity [θ] (when possible) or in mdeg, while the UV spectra were reported in molar extinction coefficient ε (when possible), or in absorbance A. The temperature was controlled through a Jasco 715 Peltier unit. Temperature controlled measurements were performed drop casting the sample into the two-pieces quartz cuvette and then sealing the edges with silicone to avoid liquid evaporation during heating. The equilibration time for each measurement was 10 min.

Cryogenic Transmission Electron Microscopy (Cryo-TEM). Cryo-TEM sample preparation and imaging was performed at the National Center for High-Resolution Electron Microscopy within Lund University. For standard cryo-TEM imaging, *i.e.* for the preparation of control samples, lacey formvar-carbon film on 200 mesh copper TEM grids (Ted Pella, Redding, USA) were glow-discharged in a Quorum GloCube system (Quorum Technologies, Laughton, UK). 4 µL of NaNAMC tubule suspension was pipetted onto the TEM grid in a Leica EM GP automatic plunge freezer (Leica Microsystems, Stockholm, Sweden) operating at 21 °C and relative humidity of >90 %, backside blotted for 2.5 s and plunged into liquid ethane. The samples were transferred onto a Fischione 2550 cryogenic sample holder and imaged on a JEOL JEM-2200FS (JEOL, Tokyo,

Japan) transmission electron microscope equipped with an omega energy filter, operating at an accelerating voltage of 200 kV. The sample temperature was kept below -174 °C during imaging. The zero-loss images were acquired on an F416.0 camera (TVIPS, Gauting, Germany) using Serial EM software (see Ref. 31) running in low-dose mode (total electron dose per acquired image <30 $e^{-}/Å^{2}$). The acquired cryo-TEM images were processed using ImageJ software (see Ref. 33).

Assembly specificity: model and calculation

In order to stress the importance of the rim topology on the specificity of the interactions, *i. e.*, the microgel assembly either at the tip or side of the tubules, we build up a simple model based on electrostatic interactions between oppositely charged interacting spheres and tubules, related to the experimental data reported in our previous paper (Cautela *et al. Small* 2018, 14, 1803215). The electrostatic potential was considered, which gave the advantage that the interaction range could be easily adjusted by considering different screening conditions. The following equation was used to describe the interactions potential

$$V(r) = \frac{q}{4\pi\varepsilon_0\varepsilon_r r} exp(-\kappa r)$$

where q, ε_0 the vacuum permittivity, $\varepsilon_0 = 80$ the relative permittivity of the dispersing medium, and κ the inverse Debye length. The effective charge of the sphere was

$$q_{eff} = \frac{sinh(\kappa R_s)}{\kappa R_s} q$$

where R_S =300 nm is the radius of the sphere and $q = +100e$ (e being the elementary charge). This corresponds to giving the sphere a uniform surface charge density. For the tube the total charge was initially homogeneously distributed at the surface as 10000 point charges per square micrometer (Fig. S1). Thereafter the calculation was repeated putting these charges only at the rims, 100 point charges per micrometer. The length of the tube was 7 µm, the line charge density was -1 e/nm which gave a total charge equal to that of the initially uniformly surface charge distributed tube. The interaction was studied for different screenings assuming either straight or helical rims at the surface of the tubules (see Figure S2a and SI). All calculations were performed at a temperature of 298.15 K. The different calculations illustrate the high specificity of the assembly at the tip for different range of interaction when the interaction sites (here charges) are located at the rims of the tubules only.

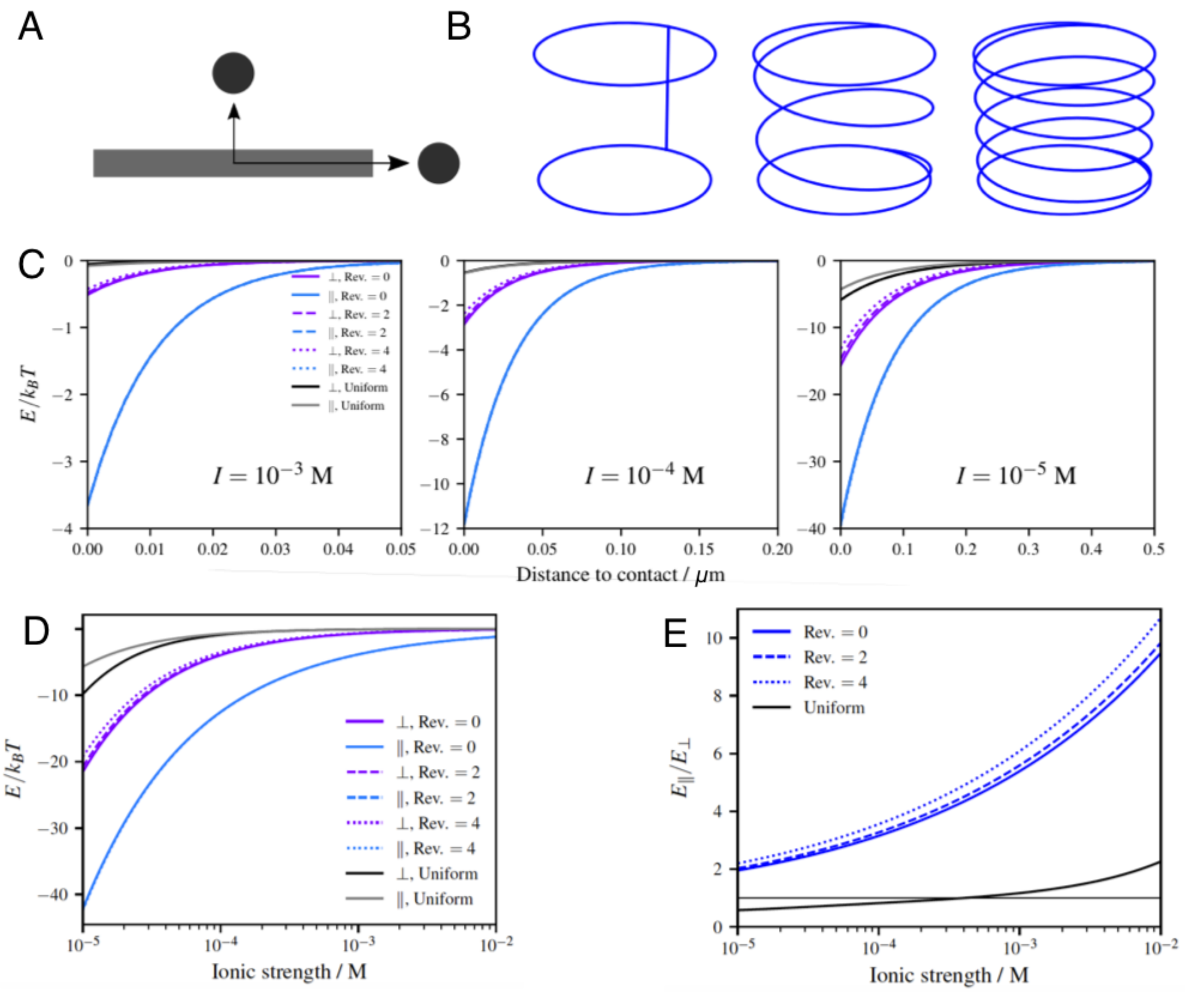

Fig. S1. Specificity of the assembly based on a simplified electrostatic model. Calculations of the electrostatic Yukawa interaction energy between a sphere and a hollow cylinder with uniform surface charge density or with the charge distributed homogeneously at the circular extremities and along a line on its surface; the cylinders have length of 3-10 μm and diameter of 450 nm while microgel have sizes ranging from 299 to 528 nm, respectively typical of the used BSD tubular structures and microgels object of the previous study[4]; the sphere either approaches along an axis perpendicular (⊥) or parallel (∥) to that of the cylinder longitudinal axis (**A**). When located along a line, the profile of the charge distribution is considered with different revolutions (rev) thereby considering straight (rev = 0) or spiral profiles with 2 (rev = 2) or 4 (rev = 4) revolutions (**B**). Calculations provided at different ionic strength show that the strength of the interaction increase by decreasing ionic strength, with a preferential interaction at the tips except for the case of hollow cylinders with uniform surface charge density (**C**). The interaction strength at contact between spheres and cylinders is shown as a function of ionic strength (**D**). Although the strength of the interaction decreases with the ionic strength, an increase of the selectivity of the interaction at the tip is observed as illustrated by the ratio of the energy at contact $E_\parallel/E_\perp$ as a function of the ionic strength (**E**).

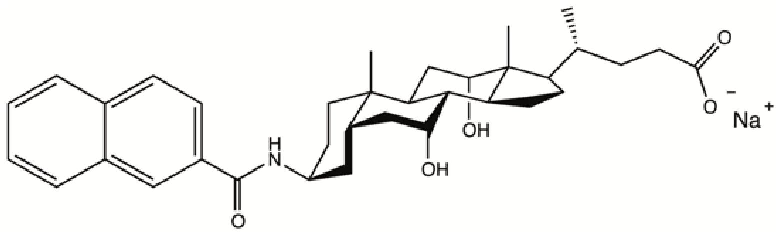

Fig. S2. Molecular structure of the sodium salt of NaNAMC.

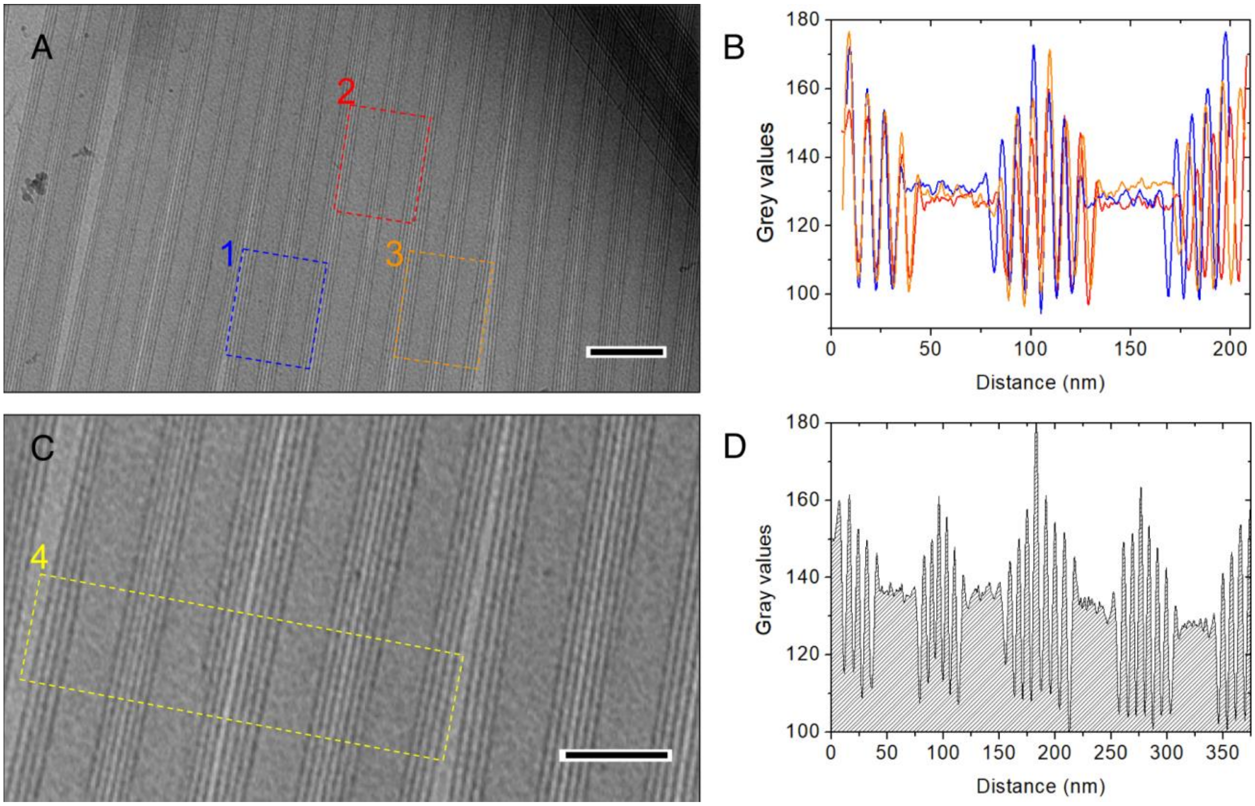

Fig. S3. Fine structural details of the NaNAMC supramolecular scrolls. Cryo-TEM micrographs of a 2.0 mM NaNAMC solution at pH 11.5 a few hours after preparation (**A** and **C**). Intensity profiles (**B** and **D**) obtained from analysis of selected areas (1-4) of the scrolls marked with dotted lines in a and c. Scale bars: 200 nm (**A**), 100 nm (**C**).

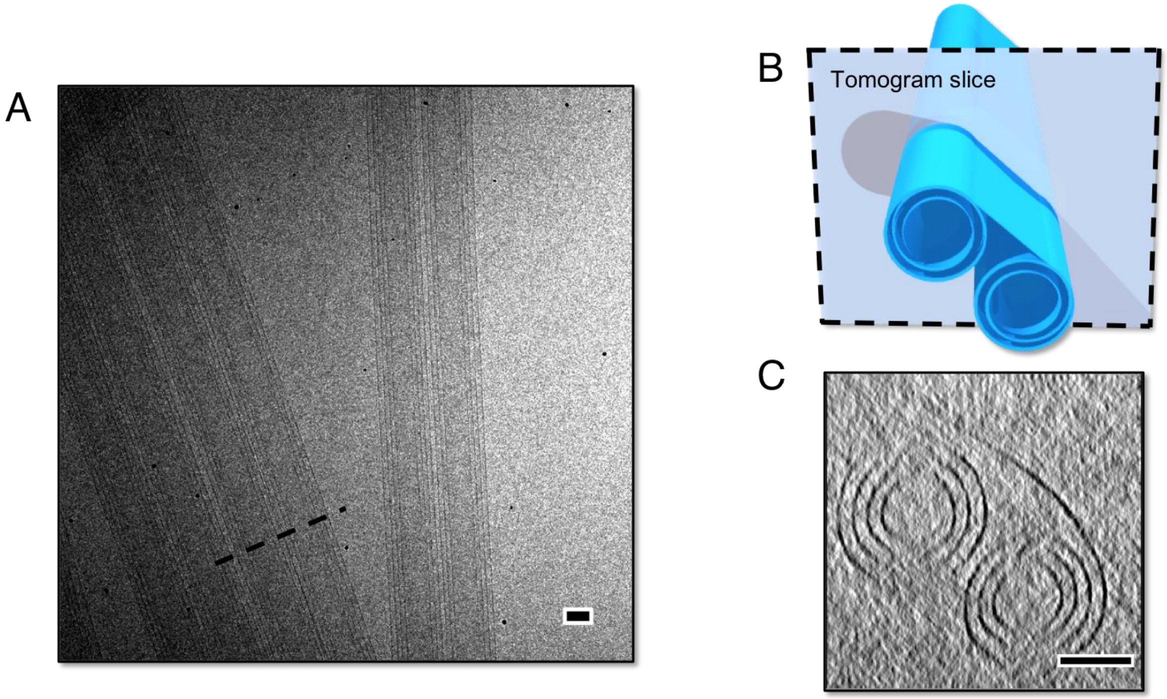

Fig. S4. Cross-sectional imaging of the NaNAMC supramolecular scrolls. Cryo-ET on a 2.0 mM NaNAMC solution at pH 11.5 allowed for 3D reconstruction of the supramolecular scroll structure. The orthogonal section of a scroll (dotted line in **A** and schematically shown in **B**, has been determined from the tomogram analysis in **C**. Scale bars: 50 nm. The whole tomogram and its analysis are shown in Video S1.

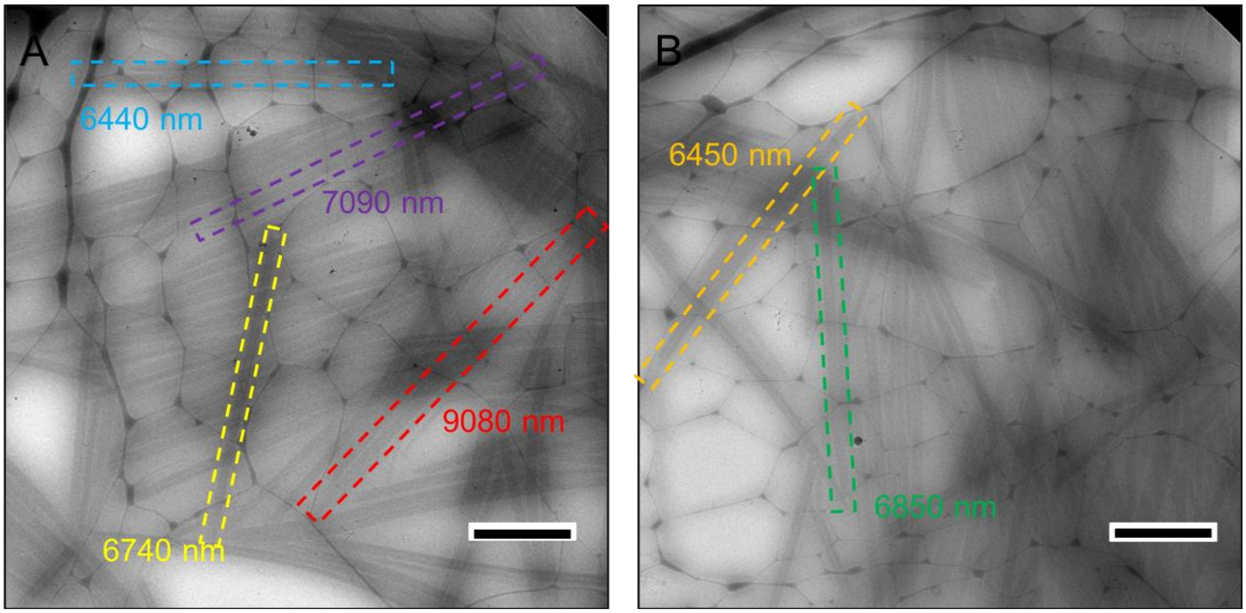

Fig. S5. Structure of the NaNAMC supramolecular scrolls. Cryo-ET micrographs of scrolls in a 2.0 mM NaNAMC solution at pH 11.5 at low magnification. Frames of colored dashed lines highlight some of the scrolls used for the statistical determination of the scroll dimensions. Scale bars: 2 µm.

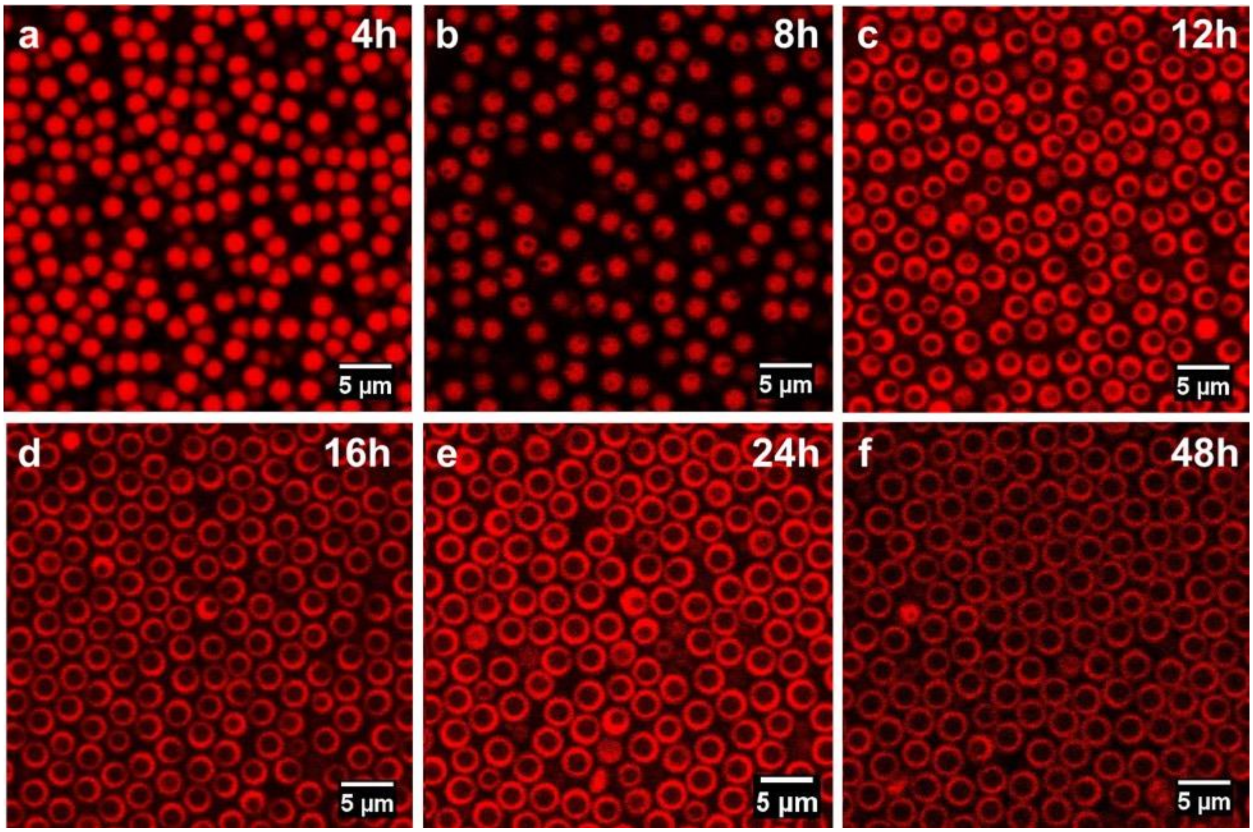

Fig. S6. Transformation from plain to hollow of the PS-PVP latex particles. CLSM micrographs showing the fluorescence emission of the particles collected during the synthesis after sedimentation at the bottom of the preparation. After 4h, the particle did not show any sign of cavitation (a) and presented a diameter of 2.06 $\pm$ 0.13 µm as determined from CLSM statistical analysis. Only after 8h, an off-centered cavity can be observed (b), which was growing over time (c-f).

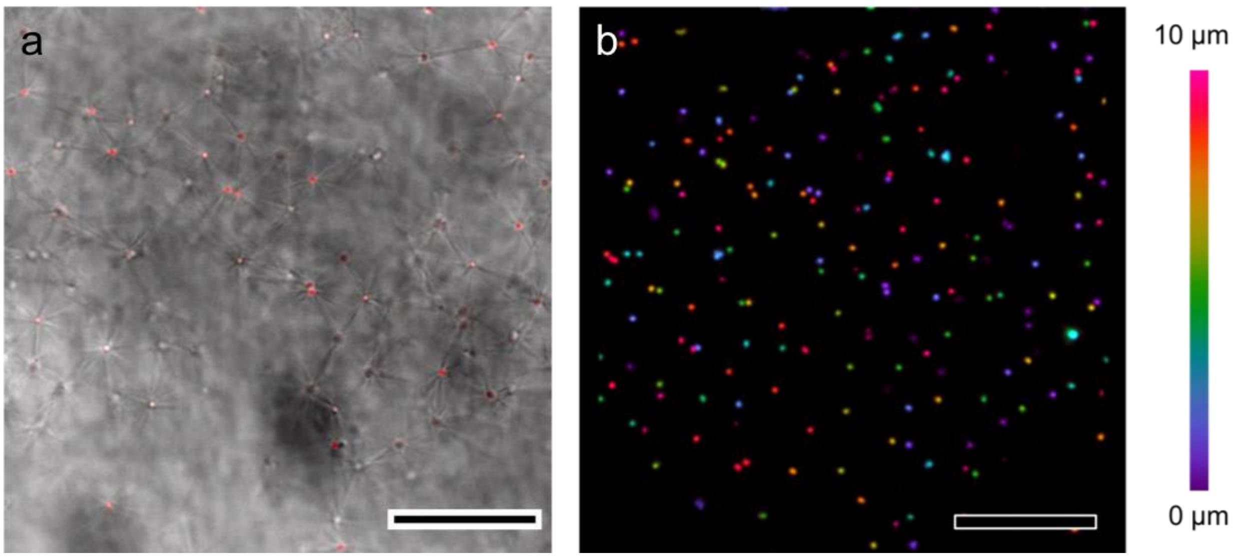

Fig. S7. Supracolloidal Atomium framework observed using cationic microgels (M+). CSLM micrograph reported as the combination of transmission and fluorescence channel of the microscope of a supracolloidal framework formed in a mixture of 2.0 · 10^{-3} wt% M+ microgels and scrolls at 2.0 mM NaNAMC concentration, at pH 11.5 and at 20 °C. The image is extracted from a *z*-stack acquisition (**A**); *z* projection of the *z*-stack showing the microgels particles color coded localization as a function of their relative *z* coordinate within the sample (**B**). Scale bar: 20 µm.

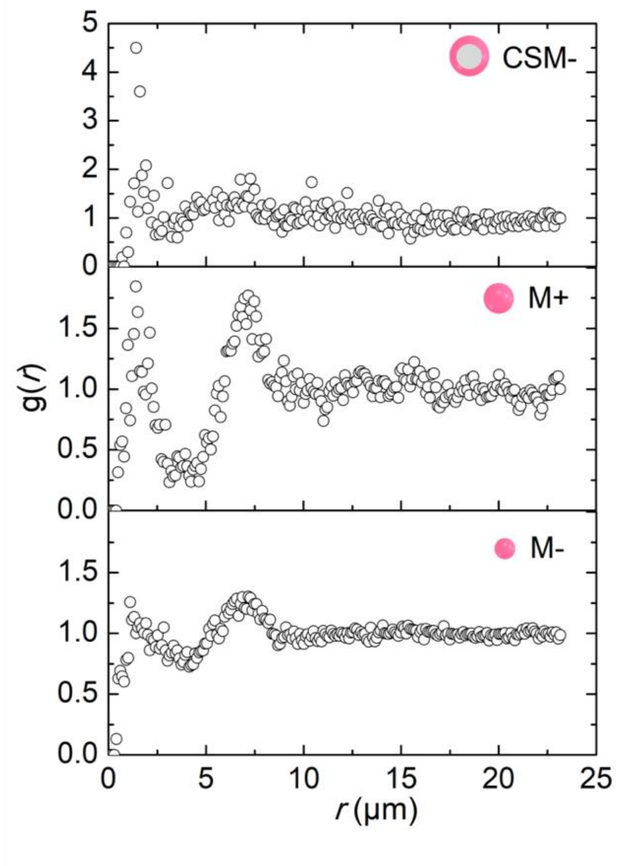

Fig. S8. Microgel-microgel correlation for frameworks formed by microgels of different sizes. Pair correlation $g(r)$ functions extracted from CLSM z-stacks performed on supracolloidal frameworks obtained in a mixture of CSM-, M+ or M- ($c_{\mu gel}$ = 5.0 · 10^{-2} wt% for the CSM- and 2.0 · 10^{-3} wt% for M+ and M-) particles and a 2.0 mM NaNAMC scroll dispersion at pH 11.5 and 20 °C.

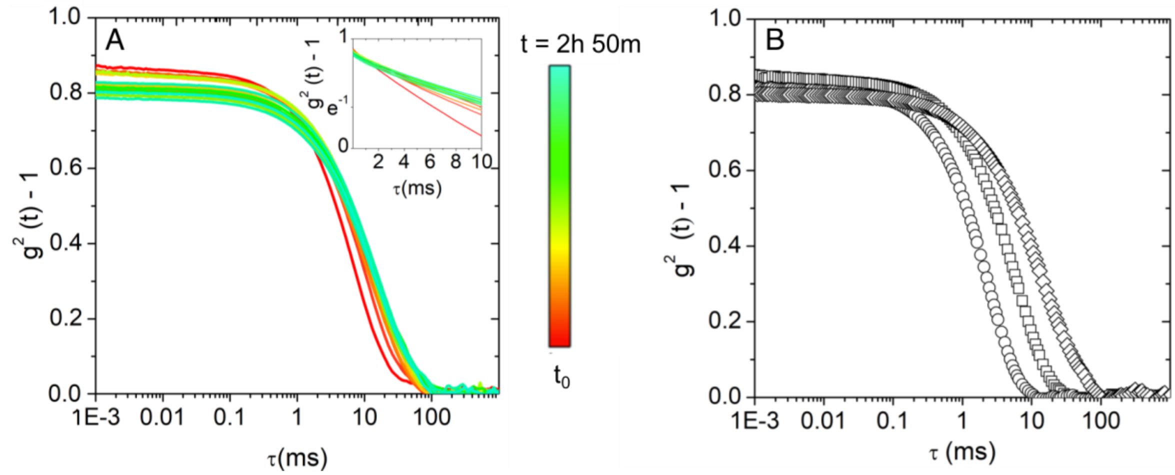

Fig. S9. Supracolloidal framework formation–kinetics by DLS. Normalized intensity autocorrelation functions for a dispersion of M+ microgels ($2.0 \cdot 10^{-3}$ wt%), interconnected by NaNAMC scrolls at pH 11.5 (2.0 mM NaNAMC). Time resolved correlation functions acquired at 90 ° every 2 min from time of mixing of the microgel and scroll dispersion and monitored for 2 h 50 min (a); comparison of the correlation curves acquired of a M+ microgel dispersion of $5.0 \cdot 10^{-4}$ wt% (circles), tubular dispersion at pH 11.5 (squares) and of a mixture of the two after framework formation (diamonds) (b).

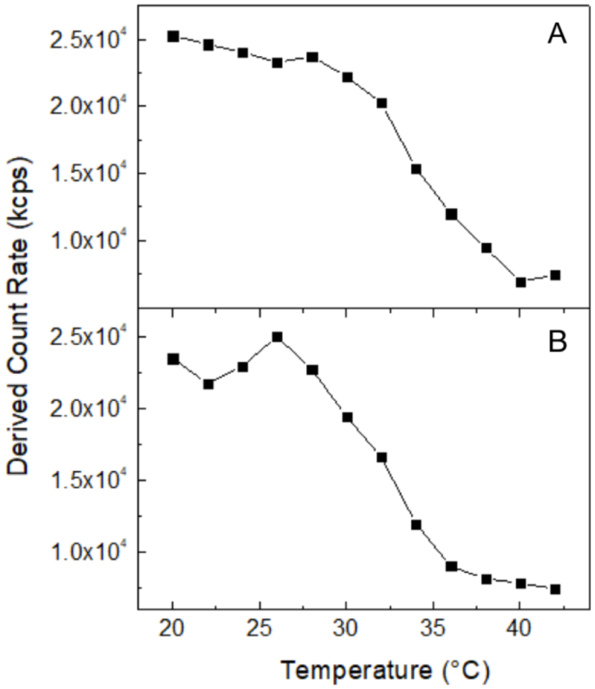

Fig. S10. Thermoresponsive behavior of the supramolecular NaNAMC scrolls. Unnormalized total light scattering intensity at 173° of a suspension of a NaNAMC solution (1.5 mM, pH 11.5) reported as a function of increasing temperature from 20 °C to 42 °C (a) and decreasing temperature from 42 °C to 20 °C (b). The measurements were performed by three consecutive measurements of 2 min each in increments of 2 °C using a Malvern Zetasizer Nano ZS instrument.

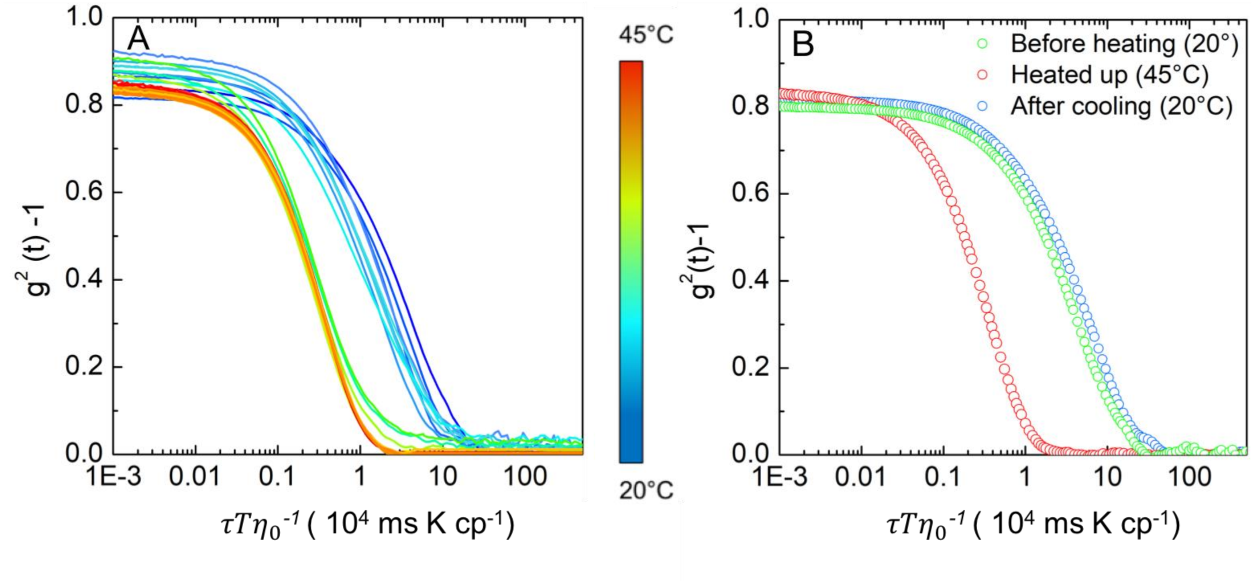

Fig. S11. Thermoresponsive behavior of the supracolloidal Atomium framework analyzed by DLS. Intensity autocorrelation functions for a mixture of 2.0 $\cdot 10^{-3}$ wt% M+ microgels and 2.0 mM NaNAMC scrolls at pH 11.5 at different temperatures (as indicated in the colored temperature scale). The pre-mixed sample was heated from 20 °C to 45 °C (red) and then measured trough stepwise cooling until the final temperature of 20 °C was reached (blue) (a). The $g^{(2)}(t) - 1$ function at 20 °C before heating (green), at 45 °C after heated from 20 °C (red) and at 20 °C after cooled down from 45 °C (b).

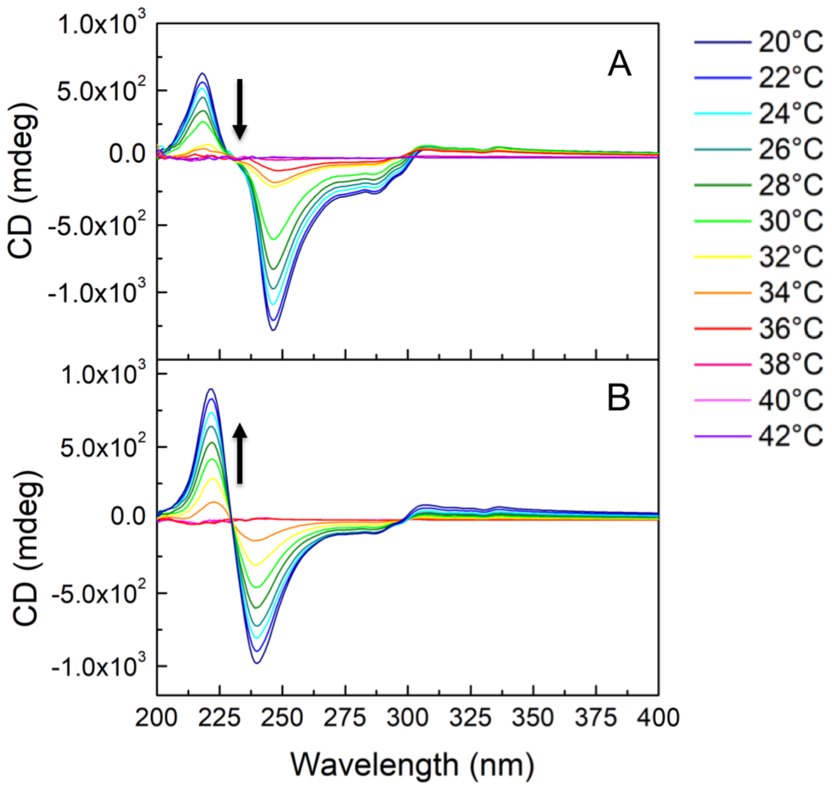

Fig. S12. Thermoresponsive behavior of supracolloidal Atomium framework analyzed by circular dichroism. Circular dichroism spectra of a mixture of 2.0 $\cdot 10^{-3}$ wt% M+ microgels and 1.5 mM NaNAMC scrolls at pH 11.5 as a function of temperature in the range 20 – 42 °C in up (a) and down scan (b).

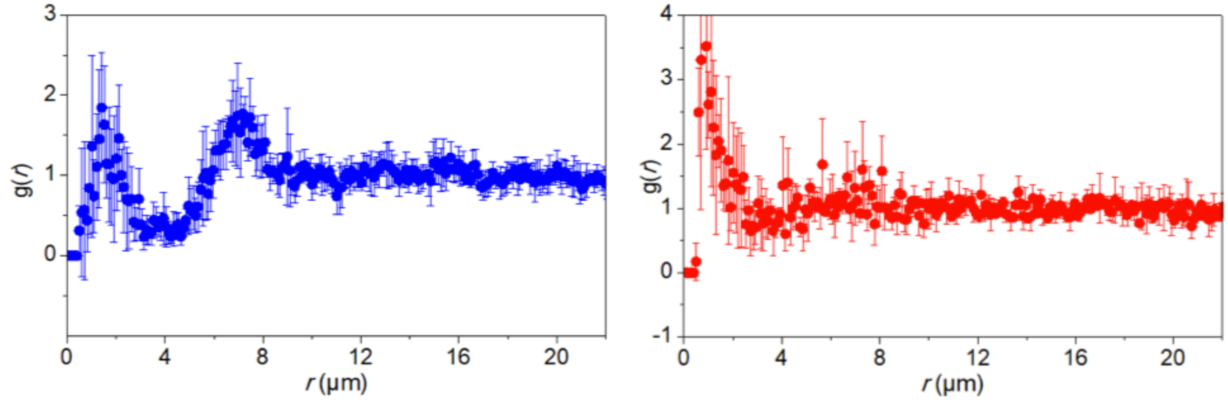

Fig. S13. Effect of framework rupture and reformation induced by temperature on the microgel-microgel correlation. Pair correlation $g(r)$ functions extracted from CLSM *z*-stacks performed on supracolloidal framework obtained for a mixture of 2.0 $\cdot 10^{-3}$ wt% M+ microgels and scrolls at 2.0 mM NaNAMC and pH 11.5: freshly prepared (blue) at 20 °C, after complete scroll dissolution at 45 °C and reformation by subsequent cooling at 20 °C (red).

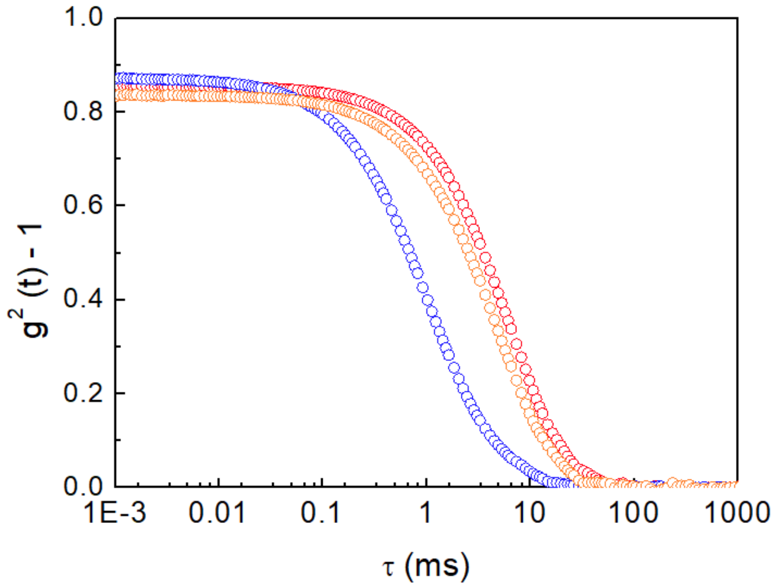

Fig. S14. pH-responsive behavior of Supra-Atomium framework analyzed by DLS. Intensity autocorrelation functions for a mixture of 2.0 $\cdot 10^{-3}$ wt% M+ microgels and scrolls at 2 mM NaNAMC, at 20 °C at different pH. The same sample was first measured at pH 11.5 (red), then at pH 7.5 (blue) (obtained by 0.1 M HCl addition), and finally at pH 11.5 (obtained by 0.1 M NaOH addition) (orange).

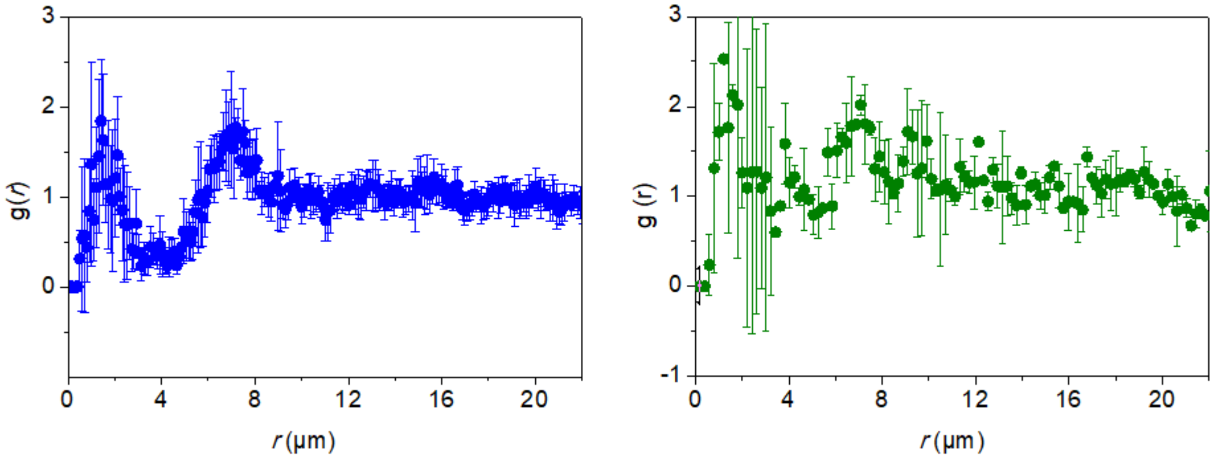

Fig. S15. Effect of network rupture and reformation induced by pH variation on the microgel-microgel correlation. Pair correlation $g(r)$ functions extracted from CLSM *z*-stacks performed on supracolloidal framework obtained for a mixture of 2.0 $\cdot 10^{-3}$ wt% M+ microgels and scrolls at 2.0 mM NaNAMC, pH 11.5 and 20 °C freshly prepared (blue) and after breaking induced acidification at pH 7.5 by 0,1M HCl addition and alkalinization to 11.5 by 0.1M NaOH addition (green).

Table S1. Particle characteristics. Spherical particles of poly(*N*-isopropylacrylamide) (PNIPAM) microgels (M+ and M-), core-shell based microgels systems consisting of a polystyrene (PS) core and shells of poly(*N*-isopropylmetacrylamide) (PNIPMAM) microgels (CSM-) and PS hollow fluorescent latex micro-particles functionalized with polyvinylpyrrolidone (PVP), (L-) used in the work. μ refers to the electrophoretic mobility measured at 20 °C.

Name	M+	CSM-	M-	L-
Composition	PNIPAM	PS/PNIPMAM	PNIPMAM	PS/PVP
Charge	Cationic	Anionic	Anionic	Anionic
Fluorophore	Rhodamine	Rhodamine	Rhodamine	Rhodamine
Core radius[1] [nm]		267[2]		908[3] (Cavity)
Radius [nm]	299[1]	528[1]	244[1]	1523[3]
μ [10^{-8}m^2/Vs]	+0.58	−1.86	−1.00	−3.07

[1] *Apparent hydrodynamic radius R_H determined by DLS measurement at 20 °C.*
[2] *Determined through TEM analysis.*
[3] *Determined through CLSM analysis.*

List of the Movies

- Movie V1. Analysis of a tubular cross section through Cryo-ET. Sequence showing 1) Cryo-ET basic functioning animation. 2) Complete tilt series (from viewing angle -60° to +60°) acquired on a 2.0 mM NaNAMC solution at pH 11.5 mixed with 5 nm AuNP fiducials. 3) tomogram (inset) showing the tubular cross-section as a function of the *x*-coordinate variation along the tubular structure longitudinal axis (yellow line).

- Movie V2. Supracolloidal assemblies of spheres and scrolls. CLSM time series of different supracolloidal assemblies obtained from the association of M- or M+ at 8.0 $\cdot 10^{-4}$ wt%, CSM- at 4.0 $\cdot 10^{-3}$ wt% and L- at 5.0 $\cdot 10^{-2}$ wt% and preformed 2.0 mM NaNAMC at pH 11.5 and 20 °C. Scale bars: 2 µm.

- Movie V3. Details of scroll-decorated M+ microgel assemblies. CLSM time series of scroll decorated M+ microgel at microgel concentration of 8.0 $\cdot 10^{-4}$ wt% and 2 mM NaNAMC at pH 11.5 and 20 °C. Scale bars: a) 5 µm, b) 2 µm.

- Movie V4. Absence of association between the scrolls and "smooth" spherical particles. CLSM time series of mixtures of NaNAMC (2.0 mM pH 11.5) scrolls with $5.0 \cdot 10^{-2}$ wt% of silica beads (green, left) and carboxylated PS particles (red, right) at pH 11.5 and 20°C. Scale bar: 5 µm.

- Movie V5. Supracolloidal cluster formation in a dilute dispersion. CLSM Time series of scroll decorated M+ microgel at microgel concentration of 8.0 $\cdot 10^{-4}$ wt% forming clusters of 3 (left), 4 (center) and 6 units (right), imaged 1h after preparation. Scale bar: 5 µm.

- Movie V6. Analysis of the 3D supracolloidal framework through CLSM. CLSM *z*-stack of a supracolloidal framework formed mixing M+ microgels at 2.0 $\cdot 10^{-3}$ wt% with 2 mM NaNAMC solution at pH 11.5 containing scrolls and 20 °C. Scale bar: 20 µm.

- Movie V7. 3D reconstruction of a supracolloidal framework from CLSM data. 3D projection reconstructing a 10 µm *z*-stack of the supracolloidal framework formed mixing M+ microgels at 2.0 $\cdot 10^{-3}$ wt% with 2 mM NaNAMC solution at pH 11.5 containing scrolls and 20°C. Particle 3D localization is color coded as a function of their relative *z* position within the reconstructed sample. Scale bar: 20 µm.

- Movie V8. Diffusion of scrolls at the microgel surface. CLSM time series reporting microgels stuck in the supracolloidal framework with free scrolls moving at the surface, in a sample obtained adding 2.0 $\cdot 10^{-3}$ wt% M+ microgels with 2mM NaNAMC solution at pH 11.5. Scale bar: 2 µm.

- Movie V9. Effect of microgel concentration on supracolloidal framework order. CLSM 10 µm *z*-stacks reporting the supracolloidal framework obtained mixing 8.0 $\cdot 10^{-4}$ wt% (a), 2.0 $\cdot 10^{-3}$ wt% (b), 4.0 $\cdot 10^{-3}$ wt% (c) or 5.0 $\cdot 10^{-2}$ wt% (d) M+ microgel with preformed NaNAMC scrolls. Scale bar: 20 µm.

- Movie V10. Reconstruction of 3D framework by computer simulation. Sequence showing 1) CLSM transmission channel 2) CLSM fluorescence channel 3) simulated network reconstruction 4) simulated network 3D reconstruction of the supracolloidal framework.

- Movie V11. Progressive slowing down of microgel diffusion in supracolloidal framework with time. CLSM Time series of the supracolloidal framework formed mixing M+ microgels at 2.0 $\cdot 10^{-3}$ wt% with 2.0 mM NaNAMC solution at pH 11.5 containing scrolls and 20 °C, at different times from preparation. Scale bar: 5 µm.

- Movie V12. Thermoresponsiveness of the supracolloidal framework. CLSM *z*-stacks showing the reversibility of the co-assembly process of the framework when the sample at 20 °C (left) is heated to 45°C and then cooled down at 20 °C (right). Scale bar: 20 µm.

- Movie V13. pH responsiveness of the supracolloidal framework. CLSM *z*-stacks and time series showing the reversibility of the co-assembly process of the framework when the pH of the sample was adjusted by controlled addition of HCl and NaOH from 11.5 (left) to 7.5 (center) and then back to 11.5 (right). Scale bar: 20 µm.
